# Effects of a Change from an Indoor-Based Total Mixed Ration to a Rotational Pasture System Combined With a Moderate Concentrate Feed Supply on Rumen Fermentation of Dairy Cows

**DOI:** 10.3390/ani8110205

**Published:** 2018-11-10

**Authors:** Julia Hartwiger, Melanie Schären, Sarah Potthoff, Liane Hüther, Susanne Kersten, Dirk von Soosten, Andreas Beineke, Ulrich Meyer, Gerhard Breves, Sven Dänicke

**Affiliations:** 1 Institute of Animal Nutrition, Friedrich-Loeffler-Institut, Federal Research Institute for Animal Health, 38116 Braunschweig, Germany; Julia.Hartwiger@fli.de (J.H.); Melanie.Schaeren@uni-leipzig.de (M.S.); sarah.potthoff.91@gmail.com (S.P.); Liane.Huether@fli.de (L.H.); Susanne.Kersten@fli.de (S.K.); Dirk.von_Soosten@fli.de (D.v.S.); Sven.Daenicke@fli.de (S.D.); 2 Clinic for Ruminants and Swine, Faculty of Veterinary Medicine, University of Leipzig, 04103 Leipzig, Germany; Melanie.Schaeren@uni-leipzig.de; 3 Institute of Pathology, University of Veterinary Medicine Hannover, Foundation, 30559 Hannover, Germany; Andreas.Beineke@tiho-hannover.de; 4 Institute of Physiology, University of Veterinary Medicine Hannover, Foundation, Bischofsholer, 30173 Hannover, Germany; Gerhard.Breves@tiho-hannover.de

**Keywords:** dairy cows, ration change, pasture, confinement, VFA, pH logger, rumen papillae morphology, non-glucogenic to glucogenic VFA ratio

## Abstract

**Simple Summary:**

In temperate climate zones, cows are in spring traditionally transitioned from a silage and concentrate- ration to a pasture-based ration. This transition requires complex nutritional and metabolic adaptions for the cow, resulting in a lower feed intake with consequences on energy metabolism. Normally concentrate feed is supplied to support the cows after transition to pasture. Depending on weather influences and growing stage, grass contains high amounts of fast fermentable carbohydrates and low amounts of physical effective fiber. In a previous trial, pasture feeding combined with low amounts of concentrate supply did not prevent an energy shortage after transition to pasture but led to changes in ruminal fermentation patterns indicating a possible risk for rumen health. However, the impact of ration change has not been extensively researched so far when moderate concentrate feed was supplied moderately in order to prevent an energy deficiency. To investigate the influences different rumen variables were documented, using continuous pH measuring devices and weekly diurnal fermentation assessments in rumen fistulated animals. Influence on rumen epithelial morphology was measured by the collection of rumen papillae biopsies and subsequent surface area, as well as histopathological analyses. With the help of this data, a greater understanding of the adaption period of the animals during transition from confinement to pasture is made possible.

**Abstract:**

In spring, transition from a total mixed ration (TMR) to pasture requires rumen adaptions for the cow. It had been shown that transition period does not necessarily mean an increased risk for subacute ruminal acidosis (SARA). After adaption to pasture, however, supplying low amounts of concentrate did indicate increased risk, but caused no adverse effects on rumen morphology and absorption capacity. The present study aimed to investigate the effect of transition, and how a supply of 4.5 kg dry matter concentrate·cow^−1^·day^−1^ during fulltime grazing influenced different rumen parameters. During a 12-week trial eleven rumen-cannulated dairy cows were observed during transition from confinement to pasture (PG; *n* = 6) and compared to cows fed TMR indoors (CG; *n* = 5). The CG stayed on a TMR based ration (35% corn silage, 35% grass silage, 30% concentrate; dry matter basis), whereas the PG slowly switched to a pasture-based ration (week 0 and 1 = TMR, week 2 = TMR and 3 h pasture·day^−1^, week 3 and 4 = TMR and 12 h pasture·day^−1^, and week 5 to 11 = pasture combined with 4.5 kg DM concentrate·cow^−1^·day^−1^). Papillae surface area decreased during transition and increased again during fulltime grazing, while the fractional absorption rate of volatile fatty acids (VFA) was not influenced. This suggests only a limited effect of papillae surface area on VFA absorption rate. Feeding changes resulted in different fermentation profiles of VFA. Changing ratio of starch to sugar during transition to fulltime grazing plus concentrate supply did not lead to lower rumen pH. In conclusion, the concentrate supply combined with high fermentable grass during fulltime grazing increased papillae surface area but did not affect absorption rate or rumen pH, so that risk for SARA was not increased.

## 1. Introduction

Vernal transition from a total mixed ration (TMR) indoor system to a full-grazing system combined with small amounts of concentrate supply was shown to result in an energy deficiency of high yielding Holstein cows as well as in complex physiological and structural adaptions of the rumen [1,2]. Cows on pasture (PG) have a higher energy requirement compared to confinement housing cows (CG), simply due to walking and an insufficient nutrient intake [3], which gives cause for an additional energy supply via concentrate feeding [4]. Especially high yielding cows need appropriate concentrate supply to maintain their milking performance in pasture based systems [5], because their dry matter intake (DMI) is lower compared to confinement housing cows [6,7,8]. Concentrate supply increases energy intake and thereby rumen fermentation and microbial protein synthesis. Bannink et al. 2008 [9] stressed the main points of rumen fermentation: composition of microbial population, type of fermented substrate, changes in ruminal microbial environment within the rumen or the characteristics of microbial metabolism. Especially in spring grass contains high amounts of fast fermentable organic matter (fOM) [10], this combined with concentrate supply results in high volatile fatty acids (VFA) production and thereby in a proliferation of rumen papillae [11] as well as low ruminal pH. Schären et al. 2016 [2] observed changes in rumen papillae surface area and after a few weeks of fulltime grazing and supply of 1.75 kg concentrate·cow^−1^·day^−1^ (dry matter basis (DM)) an increased fermentation activity up to an elevated risk of subacute ruminal acidosis (SARA). Bargo et al. 2002 [12] documented a significant decrease in rumen pH of dairy cows on pasture when more than 8 kg DM concentrate·day^−1^ was provided. In contrast, Antonio Silva et al. 2017 [13] observed—despite increasing supply of concentrate up to 6.0 kg·day^−1^—no rumen pH effect on non-lactating cows grazing on Tanzania grass pasture. Results of the present study regarding the behavior adaption [3] mirrored in an increased time spent eating for the PG, which could independent of concentrate supply lead to a higher fermentation activity. Few studies have investigated the impact of TMR and pasture fed dairy cows regarding fermentation patterns and results dealing with daily fluctuations are missing. The study of Schären et al. 2016 [2] underlines the importance of a more frequent sampling as daytime fermentation fluctuations are influenced by DMI on pasture and confinement housing. The aim of the present experiment was therefore to examine the effect of supplying an appropriate concentrate feed of 4.5 kg DM·cow^−1^·day^−1^ in a rotational grazing system on rumen papillae morphology and absorption rate of VFA as well as on the SARA risk during fulltime grazing. For this we documented changes in variables like rumen content, rumen fermentation characteristics (24 h pH course, 12 h profile of pH and VFA concentrations), VFA absorption, papillae surface area, histopathological variables and SARA risk.

## 2. Materials and Methods

Experimental work was carried out at the experimental station of the Institute of Animal Nutrition, Friedrich-Loeffler-Institut (FLI) in Braunschweig, Germany. The experiment was carried out in accordance with the German Animal Welfare Act approved by the Lower Saxony State Office for Consumer Protection and Food Safety (LAVES, Oldenburg, Germany, file number: 33.19-42502-04-15/1858), and was supported by the Ministry for Science and Culture Section of Lower Saxony (MWK), Hannover, Germany.

### 2.1. Experimental Design and Treatments

The experimental design, treatments, DMI, rations, climate data, animal performance, energy metabolism, physical activity, clinical chemistry, and total blood counts were described in Hartwiger et al. 2018 [3] and based on the setup described in Schären, et al. 2016 [1,2] except for the grazing system (rotational vs. stationary) and the amount of concentrate supplementation (1.75 kg vs. 4.5 kg DM concentrate·cow^−1^·day^−1^). 

Briefly, the experimental work started on April 18, ran about 12 weeks, and ended on July 8, 2016. The full experiment included 57 German Holstein cows (parity: 2.1 ± 1.3; 163 ± 32 DIM; 27.8 ± 1.1 kg milk·cow^−1^·day^−1^; mean ± SD; at the beginning of the trial), which were either assigned to a pasture (PG; *n* = 26) or a confinement group (CG; *n* = 31). Both groups contained rumen-fistulated animals (PG; *n* = 6; CG; *n* = 5; parity: 3.5 ± 1.9; 157 ± 32 DIM; 27.4 ± 2.5 kg milk·cow^−1^·day^−1^; mean ± SD; at the beginning of the trial). The present investigations only included fistulated cows, which were put on a comprehensive feeding experiment. 

The CG received a TMR (35% maize silage, 35% grass silage, 30% concentrate, DM-basis) throughout the trial, whereas the PG gradually transitioned towards pasture feeding (weeks 0 and 1: TMR-only, week 2: 3 h·day^−1^ on pasture, weeks 3 and 4: 12 h·day^−1^ on pasture, week 5 to 11: pasture-only plus 4.5 kg DM concentrate·cow^−1^·day^−1^). During fulltime grazing, animals spent 2 h daily in confinement for milking and concentrate supply. 

A rotational grazing system was performed, dominated by perennial ryegrass (paddock size: 1.6 ± 0.3 ha each). On average all four paddocks were covered to 79.5 ± 13.6% with grass, 7.5 ± 6.9% with herbs and 4.4 ± 7.4% with legumes (estimated pasture coverage; mean ± SD). All cows entered a paddock when sward height had reached an average of 14 cm, and left the paddock when the sward height was around 8 cm or when the visual assessment of the pasture indicated an insufficient composition. Paddock sward height was checked daily with RPM F400, (Farmworks Systems Ltd., Manawatu–Wanganui, New Zealand). The average pasture allowance amounted to 142 ± 46 kg of DM grass·cow^−1^·day^−1^ in the beginning of each grazing period and decreased on average to 87 ± 18 kg of DM grass·cow^−1^·day^−1^ after 6 days of grazing. The composition of the pasture was documented weekly from week 2 on. For this, we took pooled samples with the help of an electronic scissor in areas were the cows spent most of the time for grazing and exclusively from the upper half of the plant over one week, independent of the grazing plot. The composition of the TMR was analyzed two times. All chemical methods concerning feed analyses are based on the procedures recommended by Verband Deutscher Landwirtschaftlicher Untersuchungs- und Forschungsanstalten [14], as described in details by Schären et al. 2016 [1].

In the CG individual DMI and water intake was recorded automatically using electronic weighing troughs with ear tag detection (computerized feeding station Insentec Typ RIC, B. CF., Markenesse, The Netherlands). DMI on pasture was recorded in week 6 and 7 using the n−alkane marker method and calculated in week 2 to 11, based on modified equations according to Heublein, et al., 2017 [15], including metabolic body weight, energy corrected milk and net energy for lactation of grass, as well as using exclosure cages (described in detail in Hartwiger et al. 2018 [3]). Briefly, between week 5 until week 8 the PG was supplied with n-alkane marker, mixed into the supplied concentrate. After seven days a steady state in marker excretion is achieved and the sampling of manure starts (2 times·day^−1^) as well as sampling of pasture. However, the n-alkane concentration in manure and grass are analyzed by gas chromatography and extrapolated to DMI. Using exclosure cages the DMI can be estimated by cutting different areas on pasture: (1) reference area in size of the cage before entering the pasture, (2) area underneath the cage when rotating to the next paddock and (3) while at the same time a reference area next to the cage.

Body weight was measured twice daily, and body condition score was documented once per week. Milk yield was measured twice daily, and samples were taken twice per week for analysis. Weekly blood samples were taken for hematology, blood cell counts, and clinical chemistry.

The fistulated cows were alternately equipped with a sensor-based automatic measurement system (RumiWatch, Liestal, Switzerland; during 3 ± 1 days per week; mean ± SD) to record the time per day the animals were ruminating, eating or walking.

Weather and barn climate condition were recorded daily. The data were used to calculate the temperature humidity index (THI).

### 2.2. Rumen pH and Fluid Composition

From week 1 to week 10 rumen fluid samples were collected one day a week after morning milking on Tuesdays. Samples were taken on four different daytimes (Dt): 8:00 a.m., 12:00 p.m., 4:00 p.m., 8:00 p.m. to examine the diurnal fluctuations of the fermentable end products. 

With the help of a manual pump, rumen fluid samples were taken of the ventral sac of the rumen. Samples were cooled to 4 °C until more analyzing steps followed. 

For all time points, total VFA concentrations were determined as well as proportions of acetic acid (C2**%**), propionic acid (C3**%**), butyric acid (C4**%**), valerate (C5**%**), iso-butyrate (iC4**%**), iso-valerate (iC5**%**) as described in Geissler et al. 1976 [16], and NH_3_-N concentration was calculated using steam distillation, according to Kjedahl method DIN38406-E5-2, [17]. 

For the 1200 pm sample Lipopolysaccharide concentration (LPS) was analyzed as described elsewhere [2]. 

The non-glucogenic to glucogenic VFA ratio (NGR) was calculated as documented in Equation (1) and described in Abrahamse et al. 2008 [18].

NGR = [C2% + 2 × (C4% + iC4%) + C5% + iC5%]/[C3% + C5% + iC5%](1)

From week 1 on, rumen pH was continuously measured in the ventral rumen sac using a continuous ruminal pH measuring device (Lethbridge, Research Centre Ruminal pH Measurement System, Dascor, Escondido, CA; Penner et al. 2006 [19]. Briefly, before and after every measuring period the devices were calibrated in buffer solutions (pH 4 and 7) at 39 °C. The system recorded the pH every minute for 24 h. The PG was equipped for 5.58 ± 0.95 days with the data logger, and the CG for 3.75 ± 0.72 days (both means ± SD). For pH data interpretation the same variables as in Schären et al. 2016 [2] were calculated: logistic curve, β1 (average pH over 24 h period); β0 (pH-variation over 24 h period); and the SARA risk (threshold of 314 min at pH < 5.8·day^−1^) was worked out according to Zebeli et al. 2008 [20]. The amount of pH measurements and animals with logger differed between the weeks, that’s why a score per group and week was calculated as SARA score, mirrored in Equation (2), based on Schären et al. 2016 [2].

SARA score = [sum of (number of positive SARA observations per animal in week *i*/total number of observations per animal in week *i*)]/total number of animals in week *i* (2)

The following procedures are described in detail by Schären et al. 2016 [2]. The methods were practiced in week 1 (confinement), week 6 (2nd week of fulltime grazing plus 4.5 kg DM concentrate·cow^−1^·day^−1^) and week 10 (after 6 weeks fulltime grazing), after morning milking (5:30 a.m.) and before morning feeding (10:30 a.m.).

### 2.3. Rumen Content

In the above-announced weeks, we emptied the rumen of the fistulated cows and separated the content into fluid and solid fractions. After determining the weight, we took samples for determination of the DM and for calculation of the total rumen DM and non-DM quantity. Until analyzation the samples were kept in the freezer (−20 °C). During the experiment rumen content was kept warm in insulated barrels. After further sampling (rumen papillae collection, VFA absorption test) the rumen content was combined again and completely replaced into the rumen.

### 2.4. Rumen Papillae Collection

After evacuation papillae biopsies were taken from three different sampling sites of the rumen (*Saccus cecus caudodorsalis*, *saccus ventralis, and saccus cecus caudoventralis*). From each sampling site an equal amount of papillae was used to calculate the average papillae size of all three sampling sites. The papillae surface area was evaluated for inflammatory lesions. Lesions per papillae on the epithel site were evaluated and grouped into samples with lesions and without lesions. The average amount of lesions overall sampling sites was used for evaluation per animal.

### 2.5. VFA Absorption Test

For the VFA absorption test a physiological solution, including acetic acid (C2), propionic acid (C3) and butyric acid (C4), as well Cobalt EDTA, used as indigestible marker for the liquid phase, were filled under standardized conditions in an empty washed rumen. Details are described in Schären et al. [1,2]. After introducing the solution into the rumen, a sample was taken, another after 30 min and after 60 min of incubation time. The samples were analyzed immediately for pH and after storing in the freezer for C2, C3, and C4 concentration, as well as for the Cobalt concentration (inductively coupled plasma optical emission spectrometry). At the beginning of the test, after filling the solution into the rumen, the average pH was 5.7 ± 0.03, the average C2 concentration was 78.3 ± 8.9 mM·L^−1^, the average C3 concentration was 32.4 ± 3.8 mM·L^−1^ and the average C4 concentration was 19.4 ± 2.3 mM·L^−1^. The buffer solution was used to describe the water inflow, fractional liquid passage rate (FLPR), and fractional absorption rate (FAR) of the fatty acids mixed in the liquid.

### 2.6. Statistics

Most statistical analyses were performed using the Software SAS Enterprise Guide 7.1 (SAS Institute Inc., Cary, NC, USA). Variables recorded more than once a week were reduced to weekly means per cow before statistical analysis. For repeated measures the MIXED procedure was used combined with a restricted maximum likelihood model (REML). The model contained time (T = week), daytime (Dt) and group (G), as fixed factors as well as their interactions (G × T, G × T × Dt). To account for repeated measurements from the same cow the repeated statement was used. Best fitting covariance structures were tested using the Akaike information criterion for a finite sample size (AICC). For each treatment, least squares means were calculated, and pairwise comparisons of each week were further evaluated by multiple t-test (procedure PDIFF(*p*-values for all possible treatment differences), adjusted according to Tukey). Results were signed as significant at *p* ≤ 0.05 and a trend declared 0.05 < *p* < 0.10. Results are presented as least square means and pooled standard error of means (PSEM), unless otherwise indicated (SD = standard deviation). Multiple comparisons within experimental groups were signed by different letters, whereas group differences were presented with different symbols. Correlation coefficients between different traits were estimated using Statistica 13.0 (StatSoft Inc., Tulsa, OK, USA).

## 3. Results

### 3.1. Ration Composition and Weather Data

The chemical composition of the different rations and detailed records of the weather data are documented in Hartwiger et al. 2018 [3]. Briefly, the chemical composition of the TMR of both groups is described in Table 1. A rotational grazing system as well as concentrate supplementation (4.5 kg DM concentrate·cow^−1^·day^−1^) was practiced from week 5 on.

Temperature-humidity index (THI) was calculated daily. The outdoor THI average was 58.8 ± 6.1 being 3.9 ± 1.9 (mean ± SD) units lower compared to indoors. Periods of a mild heat stress (THI between 65 and 70 [21,22,23]) were outdoors in weeks 6 and 9 and indoors in weeks 6, 7, 9 and 11.

### 3.2. Animal Performance

For the complete herd of this study performance as well as serum clinical chemistry has already been discussed [3]. Compared to the whole group (2.1 ± 1.3) the mean number of lactations of the fistulated animals was 3.5 ± 1.9 (mean ± SD). 

Table 2 shows milk production, body condition score (BCS), body weight changes (BW) and blood glucose concentrations of the fistulated cows, representative of the rest of the herd [3].

Significant Group × Time (G × T) interactions were documented for almost all variables, except of milk fat (%), milk urea (ppm) and BCS. 

The DMI of the CG was nearly constant over the whole trial, whereas the PG showed a decrease in DMI of TMR during transition (week 2 to 5). The n-alkane method used in week 6 and 7 (week 6: 19.5 kg·day^−1^, week 7: 17.7 kg·day^−1^; data not shown) nearly matched the calculated data of total DMI. During fulltime grazing the calculated DMI was on average 3 kg lower compared to CG, resulting in a Group × Time interaction. 

The milk performance (kg) of the PG decreased by 3 kg until week 5 and by further 6 kg compared to the initial value until week 11. For the last 3 weeks the milk performance seemed to stabilize. Over time no significant differences between the groups were detected for the milk variables fat, protein and urea, whereas a variation over time was documented. 

In the CG a continuous increase in body weight of 33 kg compared to the initial value could be observed with the beginning of week 5. For the PG between weeks 0 to 6 a decrease in body weight by 45 kg was documented, followed by an increase of 35 kg, but not reaching the initial value. The changes in body condition score of the fistulated PG was not as pronounced as on group level (27 animals). Between the fistulated CG and PG only a trend over time was documented. In both groups, high variations of blood glucose concentrations were observed, resulting in a significant Group × Time interaction. The serum glucose concentration of the PG was most of the time lower compared to the CG, resulting in significant differences in week 6, 9 and 11. 

### 3.3. Rumen pH and Fluid Composition

#### 3.3.1. pH-Sensor Data

**Figure 1:**
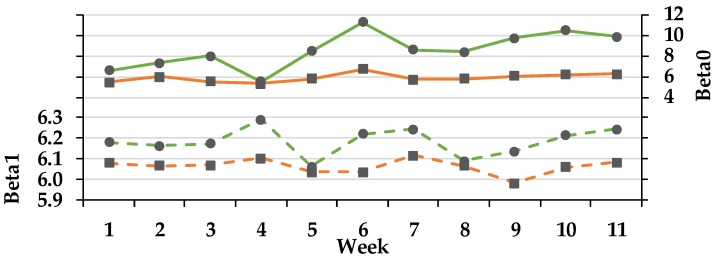
Effect of ration change from an indoor-based TMR to pasture on rumen pH. ● = pasture group (PG; green); ■ = confinement group (CG; orange). Solid line = β_0,_ the slope of the logistic curve at the inflection point illustrates the variation in rumen pH over the assessed 24-h interval (the greater the more constant; pooled SEM = 0.09); dashed line = β_1_ = inflection point of the logistic curve, representing the average pH of the assessed 24-h period (PSEM = 0.81). Significance: β_1_: group (G): *p* = 0.199, time (T): *p* = 0.348, G × T: *p* = 0.935; β_0_: group: *p* < 0.001, time: *p* < 0.01, G × T: *p* = 0.207. Logger data of week 0 of both groups are missing because of technical issues. The CG stayed on a TMR-based diet during the entire trial, while the PG was slowly introduced to a pasture-based ration: weeks 0 and 1 = TMR, week 2 = TMR and 3 h pasture·day^−1^, weeks 3 and 4 = TMR and 12 h pasture·day^−1^, week 5 to 11 = pasture and 4.5 kg DM concentrate·cow^−1^· day^−1^.

Based on the data of the pH-sensor four variables were calculated. The variables β_0_ and β_1_ as well as the time in which the pH was below the thresholds pH < 5.8 and <5.6 (min·day^−1^) were regarded to evaluate ruminal pH. The variable β_0_ describes the variation of rumen pH over the assessed 24-h interval (the greater the more constant) and β_1_ present the average rumen pH of the assessed 24-h period (Figure 1). The average rumen pH (β_1_) changed over time (*p*_T_ < 0.01) independent of the group (*p*_GxT_ < 0.935). Comparing both groups a significant difference was observed (*p*_G_ < 0.001), independent of time. 

During the trial the average pH (β_0_) of the PG was significantly higher (8.5 ± 0.2) in comparison to the CG (5.8 ± 0.3; LSmean ± SD). In addition, a time increase could be observed independent of influences of the group (*p*_T_ < 0.01). From week 5 on the curve of the PG shows an elevation of β_0_, whereas the value of the CG did not change during the trial. However, no interaction of group and time was demonstrated (*p*_GxT_ < 0.207). For the variables β_0_ and THI a positive correlation could be documented (PG: *r* = 0.801, *p* = 0.005 and CG: *r* = 0.672, *p* = 0.033). The average β_1_ value of the PG (6.0 ± 0.1) was not different from the average β_1_ of the CG (6.1 ± 0.1; LSmean ± SD). For the variable time pH < 5.8 (min·day^−1^) a tendency of a group effect was observed (PG: 133 ± 77 min/day and CG: 345 ± 84 min·day^−1^), independent of time (*p*_T_ < 0.095). The variation of the mean value (β_0_) over time gives no hint for the variation of the rumen pH over time (β_1_). A negative correlation showed up between the time the pH was below 5.8 and the variable β_0_ (*r* = −0.634, *p* = 0.0364) and β_1_ (*r* = −0.605, *p* = 0.049). Time pH < 5.6 (min·d^−1^) did not exhibit a significant group (*p*_G_ < 0.176), time (*p*_T_ < 0.410) or Group × Time interaction (*p*_GxT_ < 0.432). The pH was on average < 5.6 in the CG for 132 ± 49 min·day^−1^ and in PG for 36 ± 44 min·day^−1^ (LSmean ± SD). The calculated average SARA-score showed that the PG had a 0.15 ± 0.15 and the CG a 0.44 ± 0.07 incidence for SARA risk. During TMR and TMR plus pasture feeding (weeks 0 to 4) the highest SARA score (according Equation 2) was observed for the PG (0.44), while in week 6 to 7 and 9 to 11, during fulltime grazing plus concentrate supply, the lowest score (0.00) was documented. The CG showed with 0.5 the highest score in week 2, 5 to 7 and 10 to 11.

#### 3.3.2. DMI of Starch and Sugar and its Influence on Rumen pH

**Figure 2:**
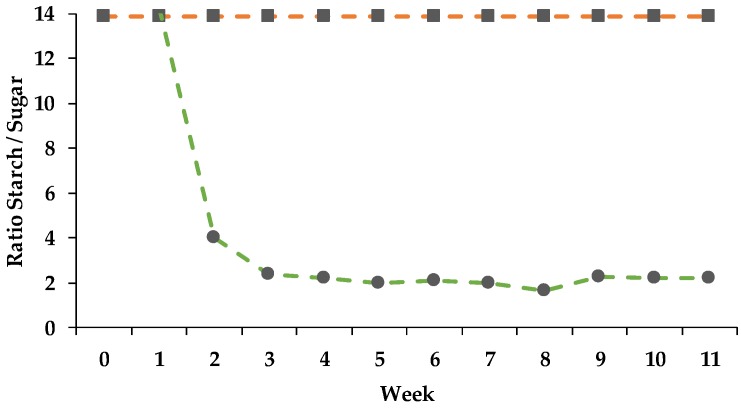
Effect of ration change from an indoor-based TMR to pasture on the ratio of starch to sugar and sugar to starch. ● = pasture group (PG, green); ■ = confinement group (CG, orange); dashed line = ratio starch to sugar; solid line = ratio sugar to starch. The CG stayed on a TMR-based diet during the entire trial, while the PG was slowly introduced to a pasture-based ration: weeks 0 and 1 = TMR, week 2 = TMR and 3 h pasture·day^−1^, weeks 3 and 4 = TMR and 12 h pasture·day^−1^, week 5 to 11 = pasture and 4.5 kg DM concentrate·cow^−1^· day^−1^.

Over the whole trial the intake of starch and sugar did not differ within the CG, which is mirrored in constant ratio of starch to sugar (Figure 2). For the PG the ratio of starch to sugar decreased, whereas the ratio sugar to starch showed the opposite pattern, as soon as the PG had access to pasture (week 2).

**Figure 3:**
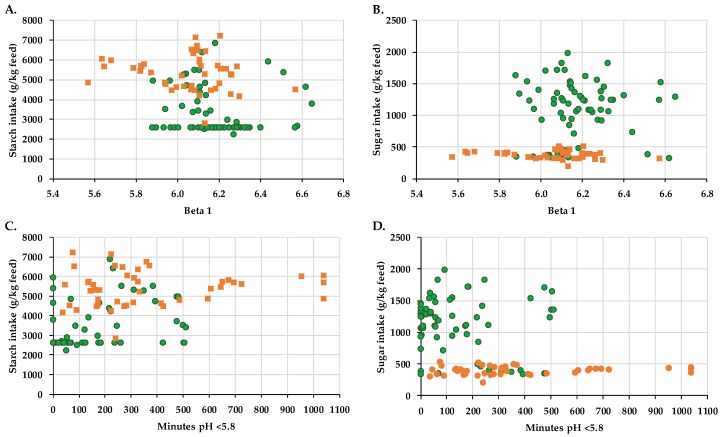
Effect of ration change from an indoor-based TMR to pasture and its influence on starch and sugar intake related to β_1_ (average pH over 24 h period) and the minutes the pH was <5.8 (according to Zebeli et al. 2008 [20]). Pasture group (PG), green; confinement group (CG), orange. (**A**). Starch intake and beta 1: r^2^ = 0.04, *p* = 0.037; (**B**). Sugar intake and beta 1: r^2^ = 0.06, *p* = 0.011; (**C**). Starch intake and minutes pH < 5.8: r^2^ = 0.12, *p* < 0.001; (**D**). Sugar intake and minutes pH < 5.8: r^2^ = 0.14, *p* < 0.001. The CG stayed on a TMR-based diet during the entire trial, while the PG was slowly introduced to a pasture-based ration: weeks 0 and 1 = TMR, week 2 = TMR and 3 h pasture·day^−1^, weeks 3 and 4 = TMR and 12 h pasture·day^−1^, week 5 to 11 = pasture and 4.5 kg DM concentrate·cow^−1^· day^−1^.

Figure 3 describes the connection between sugar or starch intake related to the average pH (β_1_), documented with the continuous measuring pH sensor, and the minutes the pH was <5.8. 

During confinement housing and transition to pasture the starch intake was higher (253 g·kg^−1^ feed) compared to fulltime grazing where the main source of starch was defined by the daily concentrate intake of 4.5 kg DM (579 g·kg^−1^ feed; Figure 3A,C). Compared to the PG the dots of the CG mirrored a higher starch intake and lower average pH values. Figure 3B shows the shift of the green dots to higher values of sugar intake (TMR in average 18 g·kg^−1^ feed, pasture on average 114 ± 45 g·kg^−1^ feed) and influences on the mean rumen pH (β_1_). Compared to the CG most of the green dots are reflecting higher pH, minimally shifting towards lower pH values. The intake of starch and sugar in relation to the minutes the pH was <5.8 shows a similar picture (Figure 3C,D). More green dots can be found at the beginning of the scale, describing the minutes the pH was <5.8, whereas the orange dots are spread over the whole scale towards higher minute values.

#### 3.3.3. VFA Concentration and Molar Proportions

**Figure A1:**
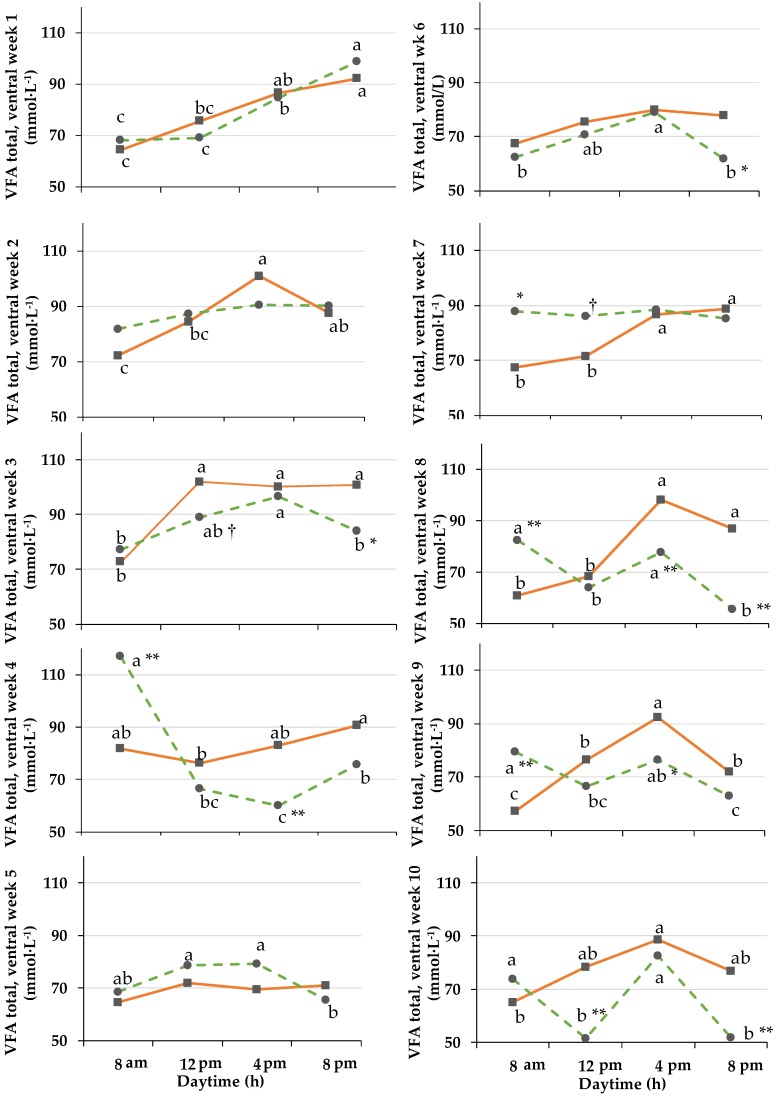
Effect of ration change from an indoor-based TMR to pasture on rumen total VFA concentration between 8 a.m. to 8 p.m. (pooled SEM = 3.3). ● = pasture group (PG, green); ■ = confinement group (CG, orange). Significance: group (G): *p* < 0.435, daytime (Dt): *p* < 0.000 time (T): *p* < 0.000, G × T: *p* < 0.000, G × T × Dt: *p* < 0.000. Different symbols indicate significant differences between groups, in particular week († *p* ≤ 0.1, * *p* ≤ 0.05, ** *p* ≤ 0.01); different letters (a–e) indicate significant differences between weeks within particular groups (*p* ≤ 0.05). The CG stayed on a TMR-based diet during the entire trial, while the PG was slowly introduced to a pasture−based ration: weeks 0 and 1 = TMR, week 2 = TMR and 3 h pasture·day^−1^, weeks 3 and 4 = TMR and 12 h pasture·day^−1^, week 5 to 11 = pasture and 4.5 kg DM concentrate·cow^−1^· day^−1^.

For the total VFA concentration a time (*p_T_* < 0.001), daytime (*p_Dt_* < 0.001), Group × Daytime (*p_GxDt_* < 0.001), Group × Time (*p_GxT_* < 0.001) and a significant interaction between Group × Time × Daytime (*p*_GxTxDt_ < 0.001) was observed. Figure A1 mirrors the course of VFA concentration per group and week during the day. In general, the VFA concentration during the day and over the weeks showed a steady increase, reaching the highest value at 8:00 p.m. For the PG this course changed with the beginning of transition and again during fulltime grazing plus concentrate supply. After 3 weeks of fulltime grazing the daily pattern looked like a two cycle system. At 8:00 a.m. and 4:00 p.m. the highest concentrations were analyzed, being significantly different to the concentrations at 12:00 p.m. and 8:00 p.m.

**Figure A2:**
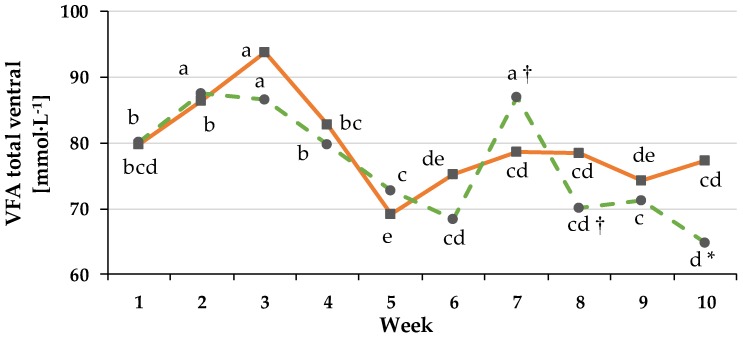
Effect of ration change from an indoor-based TMR to pasture on rumen total VFA concentration (pooled SEM = 3.3). ● =pasture group (PG, green); ■ = confinement group (CG, orange). Significance: group (G): *p* < 0.435, time (T): *p* < 0.000, G × T: *p* < 0.000. Different symbols indicate significant differences between groups in particular week († *p* ≤ 0.1, * *p* ≤ 0.05); different letters (a−e) indicate significant differences between weeks within particular groups (*p* ≤ 0.05). The CG stayed on a TMR-based diet during the entire trial, while the PG was slowly introduced to a pasture-based ration: weeks 0 and 1 = TMR, week 2 = TMR and 3 h pasture·day^−1^, weeks 3 and 4 = TMR and 12 h pasture·day^−1^, week 5 to 11 = pasture and 4.5 kg DM concentrate·cow^−1^· day^−1^.

Figure A2 shows the development of VFA concentration over the whole time period for both groups. The VFA concentration of the PG decreased continuously but not steadily decrease, whereas the decreasing concentration of the CG was followed by an increase. The weekly pattern resulted in a Group × Time interaction.

With exception of week 4 all other values for weeks significantly differed within the PG compared to week 1, where the PG received fulltime TMR. During transition to pasture (week 2 to 4) the highest VFA concentrations could be measured. During fulltime grazing plus 4.5 kg DM concentrate·cow^−1^·day^−1^ the lowest VFA concentrations were observed. In weeks 7 and 8 a tendency in difference between both groups showed up and in week 10 a significant difference as observed. The β_0_ value correlated negatively with VFA concentration (r = −0.646; *p* = 0.044).

**Figure A3:**
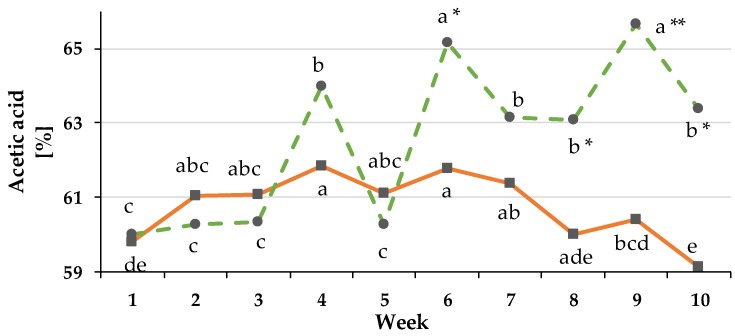
Effect of ration change from an indoor-based TMR to pasture on rumen acetic acid (C2%) proportion (pooled SEM = 0.99). ● = pasture group (PG, green); ■ = confinement group (CG, orange). Significance: group (G): *p* < 0.200, time (T): *p* < 0.000, G × T: *p* < 0.000. Different symbols indicate significant differences between groups in particular week (* *p* ≤ 0.05, ** *p* ≤ 0.01); different letters (a–e) indicate significant differences between weeks within particular groups (*p* ≤ 0.05). The CG stayed on a TMR-based diet during the entire trial, while the PG was slowly introduced to a pasture-based ration: weeks 0 and 1 = TMR, week 2 = TMR and 3 h pasture·day^−1^, weeks 3 and 4 = TMR and 12 h pasture·day^−1^, week 5 to 11 = pasture and 4.5 kg DM concentrate·cow^−1^· day^−1^.

For the molar proportion of acetate (C2%) a significant increase over time (*p_T_* < 0.001) depending on group could be observed, resulting in a GroupxTime interaction (*p_GxT_* < 0.001; Figure A3).

Between week 6 and 10 the highest proportion of C2 occurred, which led to significant differences between the groups. Furthermore, within the PG the proportion in week 6 to 10 significantly differed to week 1 and the transition period (weeks 2 and 3). In week 5 (fulltime pasture plus 4.5 kg concentrate·cow^−1^·day^−1^) C2% was significantly lower compared to week 6 to 10. The proportion in week 5 corresponded to the proportions in week 1, 2, 3.

**Figure A4:**
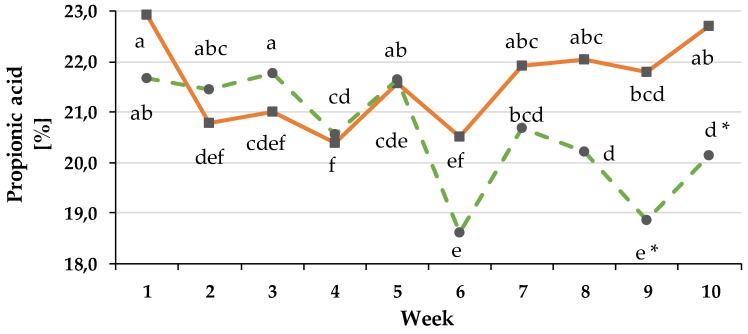
Effect of ration change from an indoor-based TMR to pasture on rumen Propionic acid (C3%) proportion (pooled SEM = 0.84). ● = pasture group (PG, green); ■ = confinement group (CG, orange). Significance: group (G): *p* < 0.372, time (T): *p* < 0.000, G × T: *p* < 0.001. Different symbols indicate significant differences between groups in particular week (* *p* ≤ 0.05); different letters (a–e) indicate significant differences between weeks within particular groups (*p* ≤ 0.05). The CG stayed on a TMR-based diet during the entire trial, while the PG was slowly introduced to a pasture-based ration: weeks 0 and 1 = TMR, week 2 = TMR and 3 h pasture·day^−1^, weeks 3 and 4 = TMR and 12 h pasture·day^−1^, week 5 to 11 = pasture and 4.5 kg DM concentrate·cow^−1^· day^−1^.

Molar proportions of propionate (C3%) decreased significantly over time (*p_T_* < 0.001), depending on group, resulting in a Group × Time interaction (*p* < 0.001; Figure A4). 

During transition, less significant changes within the PG could be observed. With the beginning of week 6 a significant decrease occurred. The C3% in weeks 6 and 8 to 10 significantly differed from weeks 1 to 3 and week 5. The C3 proportion of the PG correlated significantly with β_0_ (r = −0.686, *p* = 0.029) and blood glucose concentration (r = 0.787, *p* = 0.007). 

**Figure 4:**
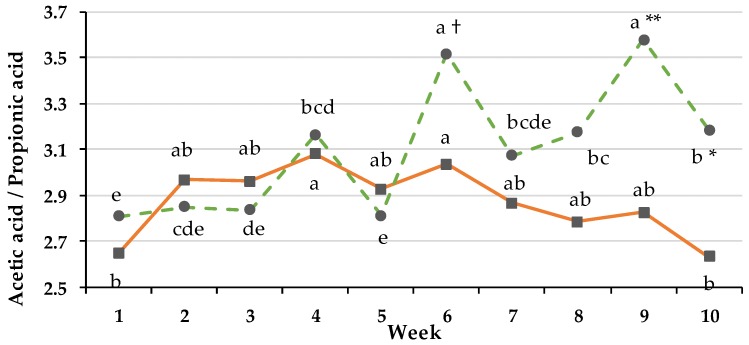
Effect of ration change from an indoor-based TMR to pasture on rumen ratio of acetate and propionate (pooled SEM = 0.16). ● = pasture group (PG, green); ■ = confinement group (CG, orange). Significance: group (G): *p* < 0.294, time (T): *p* < 0.000, G × T: *p* < 0.004. Different symbols indicate significant differences between groups, in particular weeks († *p* ≤ 0.1, * *p* ≤ 0.05, ** *p* ≤ 0.01); different letters (a–e) indicate significant differences between weeks within particular groups (*p* ≤ 0.05). The CG stayed on a TMR-based diet during the entire trial, while the PG was slowly introduced to a pasture-based ration: weeks 0 and 1 = TMR, week 2 = TMR and 3 h pasture·day^−1^, weeks 3 and 4 = TMR and 12 h pasture·day^−1^, week 5 to 11 = pasture and 4.5 kg DM concentrate·cow^−1^· day^−1^.

The acetate/propionate (C2/C3) ratio increased significantly over time (*p* < 0.001), depending on group and resulting in a Group × Time effect (*p_GxT_* < 0.001; Figure 4).

A strong and significant increase of the ratio occurred for the PG after 1 week of fulltime grazing. In week 6 a tendency in differences between both groups was observed and in week 9 and 10 the differences became significant. Weeks 6, 9 and 10 significantly differed from the weeks in confinement (week 1) and transition period (week 3 to 5).

**Figure A5:**
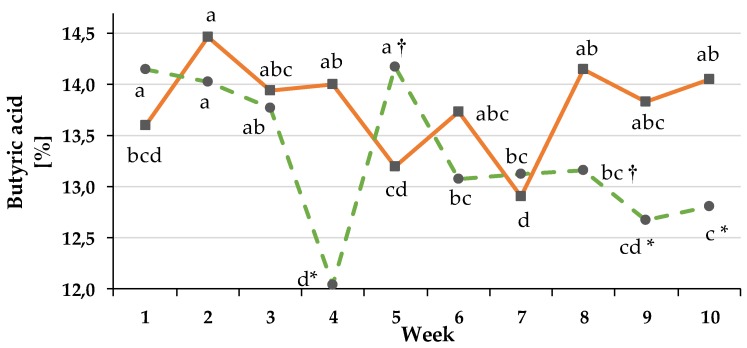
Effect of ration change from an indoor-based TMR to pasture on rumen Butyricacid (C4%) proportion (pooled SEM = 0.35). ● = pasture group (PG, green); ■ = confinement group (CG, orange). Significance: group (G): *p* < 0.168, time (T): *p* < 0.001, G × T: *p* < 0.001. Different symbols indicate significant differences between groups in particular week (* *p* ≤ 0.05, ** *p* ≤ 0.01); different letters (a–e) indicate significant differences between weeks within particular groups (*p* ≤ 0.05). The CG stayed on a TMR-based diet during the entire trial, while the PG was slowly introduced to a pasture-based ration: weeks 0 and 1 = TMR, week 2 = TMR and 3 h pasture·day^−1^, weeks 3 and 4 = TMR and 12 h pasture·day^−1^, week 5 to 11 = pasture and 4.5 kg DM concentrate·cow^−1^· day^−1^.

Molar proportions of butyrate (C4%) showed significant changes over time (*p_T_* < 0.001), depending on group and resulted in a Group x Time interaction (*p* < 0.001; Figure A5). With the beginning of fulltime grazing the Group × Time interaction showed for the PG a continuous decrease, whereas the C4% of the CG showed minor changes around the starting level. 

Only in week 4 a strong decrease with a significant difference within the PG and between both groups could be documented. Furthermore, week 5 showed the highest proportion but did not significantly differ within the PG from transition period (week 1 to 3), yet significantly changed from the sampling during fulltime grazing (week 6 to 10).

The development of the proportion of C5 showed a pronounced decrease for the PG, while the concentration of the CG slightly increased, resulting in a Group × Time interaction (*p_GxT_* < 0.01, data not shown). The same development was documented for the proportion of iC5, resulting also in a Group × Time interaction (*p_GxT_* < 0.01; data not shown). The iC4 proportion of the PG strongly fluctuated in comparison to the steady proportion of the CG, resulting likewise in a Group × Time interaction (*p_GxT_* < 0.01; data not shown).

### 3.4. Non-Glucogenic to Glucogenic Ratio

**Figure 5:**
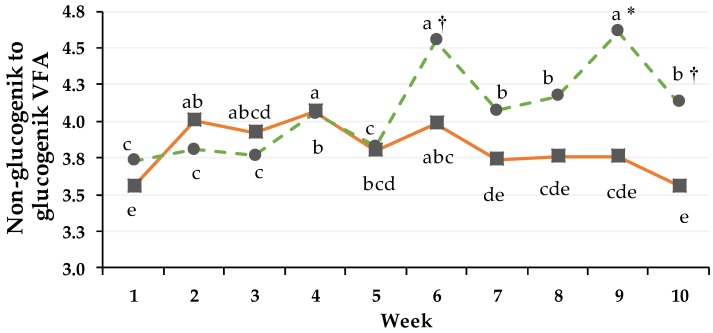
Effect of ration change from an indoor-based TMR to pasture on rumen non-glucogenic to glucogenic ratio (NGR; according to Abrahamse et al. 2009 [24] calculated weekly during week 1 to 10. ●, dashed line = pasture group (PG, green); ■, solid line = confinement group (CG, orange). non-glucogenic to glucogenic ratio. pooled SEM (PSEM) = 0.35. Significance: group (G): *p* = 0.259, time (T): *p* ≤ 0.001, daytime (Dt): *p* ≤ 0.001, G × T: *p* ≤ 0.05, G × T × Dt: *p* ≤ 0.001. Different symbols indicate significant differences between groups in particular week († *p* ≤ 0.1, * *p* ≤ 0.05, ** *p* ≤ 0.01); different letters (a–d) indicate significant differences between weeks within particular groups (*p* ≤ 0.05). The CG stayed on a TMR-based diet during the entire trial, while the PG was slowly introduced to a pasture-based ration: weeks 0 and 1 = TMR, week 2 = TMR and 3 h pasture·day^−1^, weeks 3 and 4 = TMR and 12 h pasture·day^−1^, week 5 to 11 = pasture and 4.5 kg DM concentrate·cow^−1^· day^−1^.

In PG, the proportions of C2% and C4% were higher but those of C3%, C5% and iC5% lower, resulting in a higher NGR than in CG with the beginning of week 6 (Figure 5). 

A significant interaction between Group × Time (*p*_GxT_ < 0.001) was detected, mainly caused by the larger differences in feeding regimen and chemical composition after transition. Within the PG the ratio of week 1 significantly differed from the other weeks where the DMI consisted of TMR plus pasture and later pasture plus concentrate. Significant differences between the groups were observed in week 9 (*p* < 0.05), when a pronounced ratio (as well as week 1) for the PG could be documented.

### 3.5. Rumen Variables

#### 3.5.1. Rumen Content

**Figure 6:**
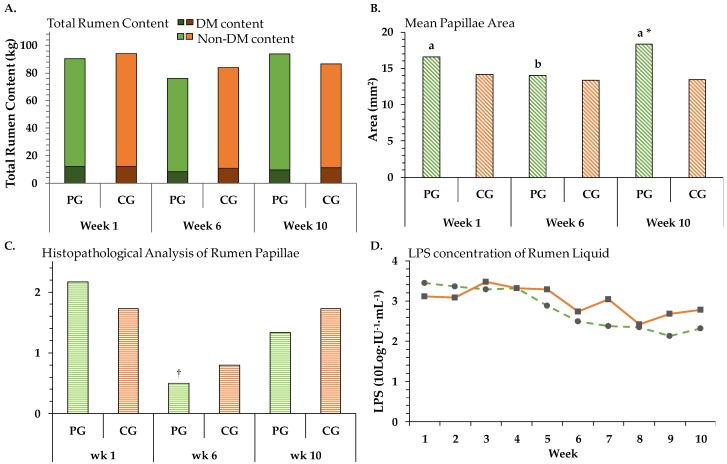
Effect of ration change from TMR to pasture on different rumen variables measured in week 1, 6 and 10 (week 1, week 6 and week 10) of the trial. Confinement group (CG, orange); pasture group (PG, green). (**A**). Rumen content. Total rumen content: pooled SEM = 6.2, significance: Group (G): *p* = 0.845, Time (T): *p* = 0.049, G × T: *p* = 0.294; Dry matter (DM) content: PSEM = 0.9, significance: Group (G): *p* = 0.116, Time (T): *p* = 0.033, G × T: *p* = 0.297; Non DM content: PSEM = 5.3, significance: G: *p* = 0.985, T: *p* = 0.047, G × T: *p* = 0.184. (**B**). Mean papillae area. PSEM = 0.01. Significance: G: *p* = 0.137, T: *p* = 0.005, Location (Loc): *p* = 0.020, G × T: *p* = 0.042; G × L: *p* = 0.500; T × L: *p* = 0.469, G × T × L: *p* = 0.565. (**C**). Histopathological analysis of papillae. Illustrated as amount of inflammations per papillae: PSEM = 0.51, significance: Group (G): *p* = 0.867, Time (T): *p* = 0.083, G × T: *p* = 0.083. (**D**). LPS concentration. ●, dashed line = pasture group (PG); ■, solid line = confinement group (CG). PSEM = 0.20. Significance: Group: *p* = 0.116, Time: *p* < 0.001, G × T: *p* = 0.158. Different symbols indicate significant differences between groups in particular week († *p* ≤ 0.1, * *p* ≤ 0.05); different letters (a,b) indicate significant differences between weeks within particular groups (*p* ≤ 0.05). The CG stayed on a TMR-based diet during the entire trial, while the PG was slowly introduced to a pasture-based ration: weeks 0 and 1 = TMR, week 2 = TMR and 3 h pasture·day^−1^, weeks 3 and 4 = TMR and 12 h pasture·day^−1^, week 5 to 11 = pasture and 4.5 kg DM concentrate·cow^−1^· day^−1^.

Total rumen content averaged 87.6 ± 14.3 kg (mean ± SD). The average DM concentration (liquid and solid phase) was 134.7 ± 12.3 g·kg^−1^ (mean ± SD). For total rumen content (*p_T_* < 0.05), DM content (*p_T_* < 0.05) and non-DM content (*p_T_* < 0.05) only a time effect could be observed (Figure 6A). Total rumen content decreased numerically from week 1 to 6 (PG: −15 kg, CG: −10 kg), whereas an increase could be observed from week 6 to 10 (PG: +18 kg, CG: +2 kg; PSEM: 6.2 kg). From week 1 to 6 a decrease of 1 kg DM content in the CG and of 4 kg in the PG could be documented (Figure 6A). Afterwards, from week 6 to 10 an increase of 2 kg in the PG could be observed, whereas the CG showed no change. For the non-DM content, a decrease of 9 kg for the CG and of 11 kg for the PG was detected from week 1 to 6. By contrast, a more pronounced increase arose from week 6 to 11 for the PG: 16 kg compared to the CG: 2 kg.

#### 3.5.2. Rumen Papillae

The average rumen papillae area (one side) was 15 ± 1 mm^2^. During the trial a Group × Time interaction (*p_GxT_* < 0.05, Figure 6B), and a single time (*p_T_* < 0.01) as well as a location (*p_Loc_* < 0.05) effect could be observed. The papillae size of the PG diminished by 15% from week 1 to 6 but expanded by 10% from week 1 to 10. Weeks 1 to 6 and weeks 6 to 10 were significantly different within the PG. The papillae size between the groups significantly differed in week 10. Minor changes occurred for the CG, a decrease of 7% from weeks 1 to 6 as well as 4% smaller papillae in week 10 compared to week 1. Histopathological analyses showed a trend of time (*p*_T_ < 0.1) as well as a trend of Group × Time interaction (*p_GxT_* < 0.1). Amount of inflammatory lesions per papillae decreased time-dependently in both groups, whereby the decrease between week 1 and 6 was with 77% more pronounced in the PG compared to the CG with 53% (Figure 6C). Afterwards, an increase of inflammation for both groups appeared, for the CG up to the initial value. In most cases diagnosis of the epithel site showed no lesions, minimally lymphoplasmacytic infiltrations or multifocal purulent pustular inflammations.

#### 3.5.3. Lipopolysaccharide Concentration in Rumen Liquid

Both groups showed a decreasing development during the trial, indicating a single time effect (*p*_T_ < 0.000; Figure 6D) without group differences. The initial value of the PG was numerical higher compared to the trial´s end. The LPS concentration of the CG peaked in week 7, and showed a higher fluctuation.

### 3.6. VFA Absorption Test with Buffer Solution

The following results rest on the experiment with buffer solution filled into the rumen after its evacuation. 

The linearity of the pH changes during incubation time was confirmed by pH assessment every 15 min, after the rumen had been filled with buffer solution (data not shown). During incubation time changes of the pH can be described with 0.033 ± 0.003 units per minute. After 60 min incubation a higher pH compared to the starting value (pH: 5.7 ± 0.03) was observed, independent of influences of group or time referred to week or its interaction (Table 3). The average concentrations of the fatty acids after filling the buffer solution into the rumen was: C2 concentration 78.3 ± 8.9 mM·L^−1^, C3 concentration 32.4 ± 3.8 mM·L^−1^ and C4 concentration 19.4 ± 2.3 mM·L^−1^. During the incubation, buffer solution concentrations of C2, C3 and C4 decreased, independent of group. Influences of time referred to week were documented.

Neither a significant single group or time effect nor their interaction could be documented for the fractional absorption rate of the solution’s fatty acids. For the parameter influx of water into the rumen (Table 3) only a time effect (*p_T_* < 0.013) occurred, independent of group. The fractional liquid passage rate increased and decreased over time *P_T_* < 0.0544) depending on group (*p_G_* < 0.018) but without an interaction of group and time. The difference between both groups consisted of the always lower value of the PG compared to the CG.

### 3.7. NH_3_-N Concentrations

**Figure A6:**
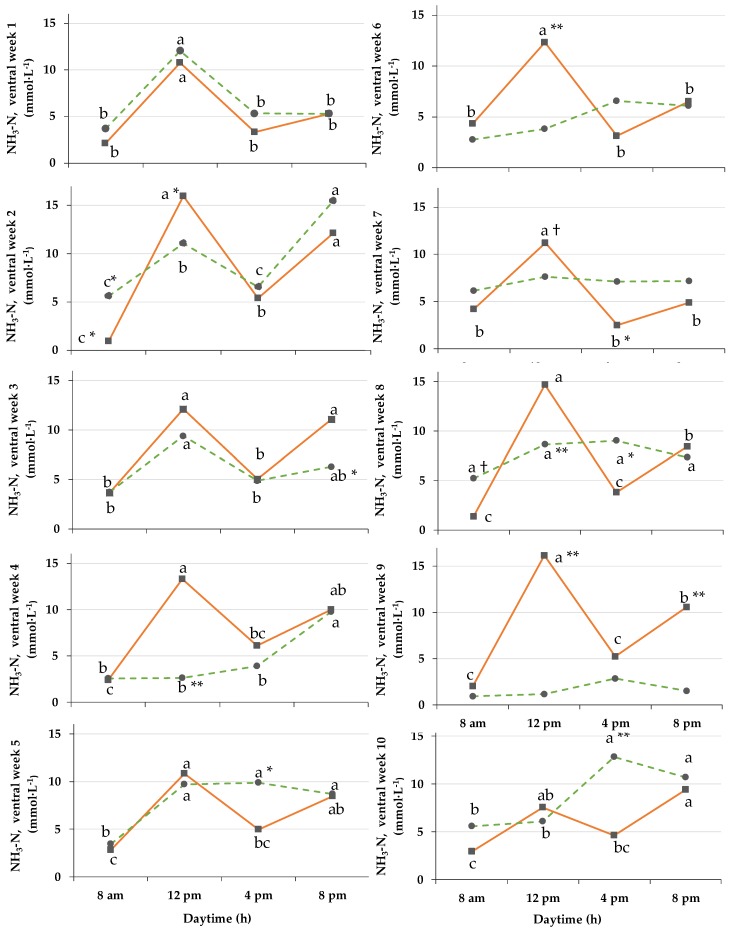
Effect of ration change from an indoor-based TMR to pasture on ammonia concentration of rumen liquid samples, measured weekly during week 1 to 10, at different time points (8:00 a.m., 1200 p.m., 4:00 p.m., 8:00 p.m.). ●, dashed line (green) = pasture group (PG); ■, solid line (orange) = confinement group (CG). Ammonia concentration (NH_3_-N), ventral. Pooled SEM = 5.3. Significance: group (G): *p* = 0.435, time (T): *p* ≤ 0.001, time point (Dt): *p* ≤ 0.001 G × T: *p*≤0.001, G × T × Dt: *p* ≤ 0.001. Different symbols indicate significant differences between groups, in particular week († *p* ≤ 0.1, * *p* ≤ 0.05, ** *p* ≤ 0.01); different letters (a–c) indicate significant differences between weeks within particular groups (*p* ≤ 0.05). The CG stayed on a TMR-based diet during the entire trial, while the PG was slowly introduced to a pasture-based ration: weeks 0 and 1 = TMR, week 2 = TMR and 3 h pasture·day^−1^, weeks 3 and 4 = TMR and 12 h pasture·day^−1^, week 5 to 11 = pasture and 4.5 kg DM concentrate·cow^−1^· day^−1^.

The NH_3_-N concentration was influenced by time (*p_T_* < 0.001), Dt (*p_Dt_* < 0.001), G × T (*p_G×T_* < 0.001), G × Dt (*p_G×Dt_* < 0.001), as well as a threefold interaction G × T × Dt (*p_GxTxDt_* < 0.001, Figure A6).

Over the course of the trial the NH_3_-N concentration of the CG showed a two-phase curve between 8:00 a.m. and 8:00 p.m., whereas the daily course of the PG began to deviate from this pattern in the third week of transition. 

The curve progression of the CG showed over all the weeks a significant increase from 8:00 a.m. to 12:00 p.m. and a significant decrease from 12:00 p.m. to 4:00 p.m., followed again by a significant increase from 4:00 p.m. to 8:00 p.m.

## 4. Discussion

In Hartwiger et al. 2018 [3], we reported the efficacy of a moderate concentrate feed supply (4.5 kg DM·cow^−1^·day^−1^) after transition from an indoor-based TMR to fulltime grazing on metabolism and production. In the current investigation, all variables concerning to performance and rumen changes were collected from 11 rumen fistulated animals integrated in the confinement group (CG; *n* = 5) or pasture group (PG; *n* = 6). However, we cannot completely exclude effects of number of parturitions on the investigated traits, since it is well known that age of the cow might have an influence on several metabolic and hematological parameters.

### 4.1. Changes of Rumen Papillae Surface Area

In the present experiment rumen papillae surface area showed a 15% reduction during transition from confinement to pasture (week 2 to 6), completed with a decreased DMI, and as a consequence a numerical degree of rumen content. The results are concomitant with the results of Schären et al. 2016 [2]. They also observed a decrease in rumen fill grade and papillae surface area during transition. In our experiment, however, during fulltime grazing combined with 4.5 kg DM concentrate·cow^−1^·day^−1^, the papillae surface increased again, namely by further 10% compared to the initial value. Papillae proliferation increases in response to the intake of high fOM, which increases papillae size and number, and hence the surface area available for VFA absorption [25]. Dieho et al. 2016 [26] detected a connection between rumen papillae surface atrophy and decreasing VFA load as a stimulus. We, also found that during transition, less DMI leads to low VFA concentrations and smaller papillae but in our trial the VFA load was still low, although the papillae increased during fulltime grazing. Ma et al. 2016 [27] found a connection between the ratio of NDF to starch and rumen epithelium structure. There, an increased ratio was associated with a decrease in expression of genes related to papillae growth. In our investigation this stimulus could only have been triggered by concentrate feed as grass does not contain starch. Because of this, we assume among other things, that the low ratio of NDF/starch influenced papillae growth. Wang et al. 2017 [28] suggests that both VFA and particle size stimulate changes in proliferation or apoptosis processes, whereby particle size is more important in regulating rumen epithelial morphology. Whether the continuous grazing system [2] or the rotational grazing system (current trial) with their two different particle sizes/pasture heights influenced papillae area can be only speculated.

### 4.2. Weekly Pattern of pH during Transition and Fulltime Grazing

Based on the results of Schären et al. 2016 [2] we had expected a VFA load combined with pH variations, resulting in an increased risk for SARA during fulltime grazing with a 2.5 times higher concentrate feed supply. Contrary to our expectation the grass combined with concentrate feeding regimen never caused a critical average ruminal pH. With the beginning of fulltime grazing plus 4.5 kg concentrate supply per day the pH variation of the PG reassembled and was more constant compared to the weeks during confinement and transition (weeks 2 to 5) as well as to the CG. This observation does not agree with other documentations [2,10,29]. According to the results of Antonio Silva et al. 2017 [13] supplementation levels up to 6 kg·day^−1^ during grazing showed no effects on the rumen pH. Bargo et al. 2012 [12] found rumen pH changes during pasture occurring when more than 8 kg concentrate (DM) were provided. The physical presentation of the grazing system could be another supposed reason for the difference in pH pattern compared to other trials. The characteristics of the practiced grazing system and the influence of environmental conditions resulted in a longer grass height (10.9 ± 2.3 cm, mean ± SD), a 28% lower sugar concentration and a 13% higher CF concentration during fulltime grazing compared to the continuous grazing system in Schären et al. [1]. Researchers already had shown the influence of feeding change on rumen microbiome, which in turn effects rumen VFA and pH [8]. During transition and fulltime grazing, the change of the ratio starch to sugar and vice versa did not influence rumen pH into low values, whereas the proportion of starch in the TMR did from time to time lead to a lower average pH in the CG. We also assume an influence of behavioral adaptations on rumen pH. The PG spent more time grazing compared to the eating time of the CG (data shown in Hartwiger et al. 2018 [3]); so that it could be presumed they would eat more evenly throughout the day. Scientists had already researched whether a higher frequent feeding system in a confinement housing system eventually prevented occurring lower pH values, but without positive results [30]. Ruminal pH varies during the course of a day and is particularly influenced by the amount of fermentable carbohydrate per meal. The study of Filho et al. 2012 [31] found that grazing time does not influence ruminal fermentation, which depends on the changes that occur when different sward layers are grazed. Structural properties depending on particle length of the feed affect chewing activity combined with saliva production [32]. This probably prevented a pronounced decrease of rumen pH in the current trial. Based on the results of the continuous pH measuring devices and calculated SARA score, no increased SARA risk was observed for the PG, whereas the SARA score for the CG showed an increased risk in week 2, 5 to 7 and 10 to 11. The low degree of inflammation of rumen papillae supports this view. In conclusion, individual variations influenced rumen pH, which underlines the importance of time needed for adaption without overloading rumen metabolism. Over time the LPS concentration developed almost equally between groups, indicating only an influence of climate conditions. Nevertheless, the PG showed a numerical decrease of LPS concentrations, whereas the course of the CG showed higher variations between the weeks, which could be a reflection of feeding type (starch, concentrate). 

### 4.3. Development of Rumen Fill Grade, Consequences on VFA Production

The pH development of the PG could also be a reflection of rumen fill grade. From week 1 to 6 we observed a numerically decrease of 16% in rumen fill grade of the PG, followed by an increase of 3% compared to the initial value. Schären et al. 2016 [2], had associated the decrease of rumen fill with a decrease in DMI rather than an increased turnover rate of grass compared with TMR. Our data confirms this, as it indicates a reduced fermentation activity. The turnover rate describes the net result of oral inflow and aboral outflow as well as absorption and secretion. At the end of the trial, for the PG a numerical increase especially of rumen liquid (plus 7% compared to the initial value) could be observed, probably due to the higher water content in fresh grass compared to silage. Melo et al. 2013 [33] had found that cows with a less pronounced acidic rumen pH have a greater inflow of water and a more developed rumen mucosa connected with a higher efficient VFA absorption. Our VFA absorption test showed a numerical higher influx of water for the PG, compared to the initial phase. Ueda et al. 2016 [34] observed that the WSC content of grass in the morning was an important factor that limits morning grazing time, and hence presumably herbage intake. Maybe the experiment of rumen evacuation would have been more exact in the dusk when the DMI is higher compared to other periods during the day [35,36]. However, we assume a low ruminal pH throughout the trial was not observed due to the low intake rate of grass (being normally a fast-fermentable substrate; in weeks 5 to 11 average grass intake was 13.3 kg) and its changes of chemical composition [3].

Differences in the chemical composition of grass over the course of the trial as well as between the continuous grazing system [1,2] and the rotational grazing system could also be documented in fermentation pattern of the different molar proportions of VFA. Compared to the indoor housing the average sugar intake during fulltime grazing was three times greater (weeks 0 and 1: 380 g vs. week 5 to 11: 1183 g), while the NDF intake was 7% higher (weeks 0 and 1: 7586 g vs. week 5 to 11: 8152 g) within the PG. Related to the average DMI (week 0 to 11: 21.1 kg·day^−1^ (DM); week 5 to 11: 17.8 kg·day^−1^ (DM)) the proportion of NDF to DMI was greater (45%) during fulltime grazing compared to TMR feeding. Based on the results of chemical composition of the feeding system, we observed an increase of C2% and a decrease of C3% and C4%. For the PG the change of feeding was related to a higher NGR value (non-glucogenic to glucogenic VFA ratio, [24]; Equation 2), mainly caused by the larger differences in feeding regimen and chemical composition after transition. The increase in NGR was influenced by the pronounced decrease of C3% and strong increase of C2% and C4%, as main fatty acids. The decrease of the proportion of C3 and its positive correlation to blood glucose concentration would explain why the blood glucose concentration (r = 0.787, *p* = 0.007) was most of the time lower compared to that of the CG. Leng et al. 1967 [37] observed that in the liver more than half of the synthesized glucose was converted from C3, which underlines the importance of C3 regarding blood glucose level. In contrast, De Menezes et al. 2011 [38] found a high abundance of propionate producing bacteria in cows fed pasture. The influences on molar proportions of VFA are also a result of the adaption of rumen microbiota throughout dietary and environmental condition changes. Schären et al. 2017 [8] illustrated that the rumen microbiota requires days to weeks to adapt to new conditions.

The change in feeding system is mirrored in total VFA concentrations, which decreased continuously with increasing time on pasture. Apart from that, we also assume the influence of temperature on the VFA concentration. It is thought that animals reduce feed intake to decrease metabolic heat production [1,39]. At noon when temperatures reach the highest level, most of the time the lowest VFA concentration could be documented in both groups, especially in week 9 and 10 when the THI got higher than 60. Taweel et al. 2004 [40] documented diurnal fluctuations of VFA. They recorded the highest concentrations especially in the dusk. In the present trial, with the beginning of week 8 a two-phase curve could be documented for the PG, reaching the highest total VFA concentrations at 8:00 a.m. and 4:00 p.m. At 6:30 a.m. and 3:00 p.m. the PG was supplemented with concentrate, which could explain the two phase curve. Filho et al. 2012 [31] showed in their grazing study the effect of young and old sward characteristics on ruminal fermentation. While the chemical composition of the feed of the CG was nearly constant, the chemical composition of grass changed during the day. Plant sugar was stored and used for respiration during daytime. A higher sugar concentration during the night combined with a higher DMI during dusk could be another reason for the high VFA concentrations at 8:00 a.m. Because of this, rotation to another plot should always be practiced in the afternoon better than in the morning. Rumen fluid collection at night would give further hints on VFA concentration and grazing behavior. During transition to another plot we observed a higher rumen pH in the last 24 h before rotation, compared to the daytime after transition when the pH was 0.5 pH-units lower. These results confirm our observation of a higher intensity of grazing after rotation increasing the VFA production and as a consequence the short time decrease of rumen pH.

### 4.4. Development of NH_3_-N Concentration in Rumen During Ration Change and Fulltime Grazing

As already reported in Schären et al. 2016 [2] the NH_3_-N concentration as well as serum urea and milk urea concentrations increased more or less as soon as the PG had access to pasture [3]. The pronounced elevation of NH_3_-N concentration during fulltime grazing could not be documented because of the low average concentration of CP compared to confinement feeding (TMR: 159 g·kg^−1^ vs. (pasture week 5 to 11): 138 g·kg^−1^). During fulltime grazing the daily pattern of NH_3_-N concentration showed no pronounced changes, as expected in Schären et al. (2016 b). Filho et al.,2012 [31] observed that young and old swards lead to a different daily pattern of ruminal fermentation except of NH_3_, while offering different sward types resulted in diurnal fluctuations of NH_3_. Carruthers and Neil [41] found that the supplementation of non-structured carbohydrates (NSC) lead to reduced ammonia concentrations, suggesting that NSC was limiting microbial synthesis on high nitrogen pasture (CP: 176 g·kg^−1^ (DM)).

## 5. Conclusions

Transition from confinement to pasture plus moderate concentrate supplementation of 4.5 kg DM·cow^−1^·day^−1^ showed changes in rumen fermentation variables but less pronounced compared to other trials. However, unlike papillae surface area, the VFA fractional absorption rate was not affected by transition from confinement to pasture or fulltime grazing. This suggests only a limited effect of papillae surface area on VFA absorption rate. We could also show that a rotational grazing system compared to a trial with a continuous grazing system resulted in different VFA fermentation profiles. Our results additionally demonstrate the influence of transition feeding and fulltime grazing on the pattern of ruminal fermentation during the day. The characteristics of the rotational grazing system, like higher grass height and higher fiber concentrations, led to an increase in molar proportions of acetate, whereas a decrease of propionate and butyric acid was observed. Continuous rumen pH and LPS concentration assessment at no time revealed an increased risk for SARA despite concentrate feeding during fulltime grazing. Changing ratio of starch to sugar during transition showed no critical influence on rumen pH for the PG. These results were confirmed by a less pronounced degree of inflammation of papillae surface area. Further studies are needed to describe rumen fermentation patterns during a more frequent rotation to a new paddock and its result on SARA risk as well as during night. Further research needs to be done to describe gene associated adaptions in rumen papillae in more detail. 

## Figures and Tables

**Table 1:** Chemical composition of experimental diets.

Type of Feeding	Item (g/kg of DM, unless otherwise noted)											
	DM (g·kg^−1^)	Ash	CP	uCP	NEL*	Sugar	Starch	RNB	CF	NDF_OM_	ADF_OM_	EE
TMR CG/PG ^1^	471 ± 6	66 ± 0	158 ± 1	152 ± 0.5	6.8 ± 0	18 ± 0	253 ± 2.5	1.1 ± 0.3	184 ± 0	363 ± 0	205 ± 1.5	37 ± 0.5
Pasture PG	174 ± 23	94 ± 9	188 ± 19	142 ± 7	6.2 ± 0.3	114 ± 45	--	6.2 ± 1.9	231 ± 34	471 ± 40	254 ± 37	41 ± 5.8
Concentrate PG	899	108	93	148	7.6	27	579	8.9	31.5	124	42	30

^1^TMR = total mixed ration; CG = confinement group; PG = pasture group; DM = dry matter; uCP = utilizable crude protein; *NEL = net energy lactation (MJ·kg^−1^ of DM); RNB = ruminal nitrogen balance; CF = crude fiber; NDF_OM_ = neutral detergent fiber; ADF_OM_ = acid detergent fiber; EE = ether extract. NDF and ADF were expressed without residual ash and are therefore referred to as NDF_OM_ and ADF_OM_.

**Table 2:** Effect of a ration change from TMR to pasture on animal performance.

Variable ^1^	Group ^2^	Week												*p*-Value			
		0	1	2	3	4	5	6	7	8	9	10	11	PSEM ^3^	G	T	G × T
DMI (kg·d^−1^) ^4^	CG	19.1	19.9	21.4	20.3	20.8	21.5	22.5	21.9	21.2	21.8	22.8	20.4	1.2	0.079	0.05	<0.01
DMI (kg·d^−1^) ^5^	PG	20.7	21.2	21.5	19.8	15.6	17.7	18.9	18.8	18.5	16.3	17.5	17.2				
Milk yield	CG	29.3	28.3	27.5	28.8	28.9	28.9	28.6	28.1	28.4	28.0	27.3	27.2	2.7	0.523	<0.01	<0.01
(kg·d^−1^)	PG	29.1	29.4	29.8	29.4	26.0	27.1	25.5	25.1	24.7	22.6	23.8	22.8				
Milk fat	CG	4.3	4.5	4.8	4.3	4.4	4.2	4.4	4.4	4.1	4.3	4.5	4.2	0.25	0.170	0.533	0.141
content (%)	PG	3.6	4.1	3.5	3.9	4.4	4.0	4.0	4.1	4.0	3.9	4.1	4.0				
Milk protein	CG	3.2	3.3	3.0	3.2	3.2	3.1	3.1	3.2	3.3	3.2	3.3	3.4	0.1	0.635	<0.01	0.075
content (%)	PG	3.2	3.2	3.3	3.2	3.0	3.1	3.0	3.1	3.2	3.1	3.2	3.3				
Milk urea	CG	129	139	142	183	211	166	166	177	179	173	180	185	17	0.181	<0.001	0.098
ppm	PG	160	159	174	210	218	195	214	202	198	170	258	168				
Body weight	CG	662	663	658	656	651	660	660	670	670	681	693	695	40	0.907	<0.001	<0.001
(kg)	PG	685	683	670	664	651	651	640	646	645	658 ^6^	670	675				
Body condition	CG	2.9	2.9	3.0	3.0	3.1	3.1	3.0	3.0	3.1	3.1	3.1	2.9	0.2	0.955	0.069	0.878
score (scale 1-5)	PG	2.9	3.0	3.0	3.0	3.2	3.1	3.0	2.9	3.0	3.0	3.1	3.0				
Blood glucose	CG	59.4	63.3	64.6	58.3	63.1	63.6	59.1	51.7	61.1	62.2	47.6	54.5	2.8	0.143	<0.001	<0.01
(mg·dL^−1^)	PG	56.7	60.3	60.4	56.5	56.3 ^†^	57.6	48.8 *	55.3	58.6	52.7 *	58.3 ^†^	46.9 *				

Different symbols indicate significant differences between groups in particular week (^†^ *p* ≤ 0.1, * *p* ≤ 0.05, ** *p* ≤ 0.01).^1^ Dry matter intake (DMI), Body weight (BW), BCS (Body condition score), ppm (parts per million); Material and Methods are described in detail in Hartwiger et al. [3] ^2^ Fistulated animals only (PG; *n* = 6; CG; *n* = 5); The CG stayed on a TMR-based diet during the entire trial, while the PG was slowly introduced to a pasture-based ration: weeks 0 and 1 = TMR, week 2 = TMR and 3 h pasture·day^−1^, weeks 3 and 4 = TMR and 12 h pasture·day^−1^, week 5 to 11 = pasture and 4.5 kg DM concentrate·cow^−1^·day^−1^. ^3^ PSEM = pooled standard error of the mean; G = group, T = time ^4^ DMI was documented by means of weighing troughs. ^5^ DMI in weeks 0 and 1 was documented by means of weighing troughs, DMI in week 2 to 5 was documented by means of weighing troughs plus calculated DMI on pasture (Method described in Hartwiger et al. [3]), DMI on pasture in week 5 to 11 was calculated as described, including 4.5 kg DM concentrate·cow^−1^·day^−1^. ^6^ Because of technical problems the BW of week 9 was assumed as the mean of weeks 8 and 10.

**Table 3:** Effect of a ration change from TMR to pasture on variables documenting VFA absorption.

		Week				*p*-Value		
Variable	Group ^1^	0	6	10	PSEM ^2^	Group	Time	G × T
Buffer solution pH 60 min	CG	7.01	6.91	7.07	0.02	0.753	0.105	0.241
	PG	6.9	6.96	7.01				
Buffer solution C2 60 min (mmol·l^−1^)	CG	35.3	41.8	41.9	2.2	0.374	<0.001	0.547
	PG	30.8	39.4	44.4				
Buffer solution C3 60 min (mmol·l^−1^)	CG	11.9	14.2	14.5	0.91	0.273	<0.001	0.639
	PG	10.3	13.2	14.8				
Buffer solution C4 60 min (mmol·l^−1^)	CG	5.4	6.7	7.1	0.56	0.446	<0.001	0.866
	PG	4.9	6.3	6.9				
Fractional absorption rate C2·h^−1^	CG	0.35	0.33	0.37	0.04	0.718	0.417	0.380
	PG	0.44	0.34	0.32				
Fractional absorption rate C3·h^−1^	CG	0.56	0.52	0.56	0.05	0.478	0.425	0.324
	PG	0.64	0.54	0.54				
Fractional absorption rate C4·h^−1^	CG	0.8	0.75	0.75	0.06	0.407	0.404	0.868
	PG	0.88	0.76	0.79				
Influx of water (L·h^−1^)	CG	7.3	8.6	10.3	0.98	0.57	0.013	0.247
	PG	6.9	11.0	9.6				
Fractional Liquid passage rate·h^−1^	CG	0.33	0.45	0.41	0.07	0.018	0.054	0.742
	PG	0.12	0.34	0.29				

^1^ Dry matter intake: CG (week 0 to 11) and PG (weeks 0 and 1) DMI from TMR only, PG: (week 2) DMI from TMR and 3 h pasture, (weeks 3 and 4) DMI from TMR and 12 h pasture, (week 5 to 11) fulltime grazing plus 4.5 kg DM concentrate·cow^−1^day^−1^. ^2^ PSEM = pooled standard error of the mean; G = group, T = time.
